# Barium Titanate Nanoparticles Sensitise Treatment-Resistant Breast Cancer Cells to the Antitumor Action of Tumour-Treating Fields

**DOI:** 10.1038/s41598-020-59445-x

**Published:** 2020-02-13

**Authors:** Yi Na Yoon, Dae-Sik Lee, Hyung Ju Park, Jae-Sung Kim

**Affiliations:** 1 Division of Radiation Biomedical Research, Korea Institute of Radiological and Medical Sciences, Seoul, 01812 South Korea; 2 Radiological and Medico-Oncological Sciences, University of Science and Technology, Daejeon, 34113 South Korea; 3 Electronics and Telecommunications Research Institute, Daejeon, 34129 South Korea

**Keywords:** Nanoparticles, Cancer prevention, Bionanoelectronics

## Abstract

Although tumour-treating fields (TTFields) is a promising physical treatment modality based on disruption of dipole alignments and generation of dielectrophoretic forces during cytokinesis, not much is known about TTFields-responsive sensitisers. Here, we report a novel TTFields-responsive sensitiser, barium titanate nanoparticles (BTNPs), which exhibit cytocompatibility, with non-cytotoxic effects on breast cancer cells. BTNPs are characterised by high dielectric constant values and ferroelectric properties. Notably, we found that BTNPs sensitised TTFields-resistant breast cancer cells in response to TTFields. In addition, BTNPs accumulated in the cytoplasm of cancer cells in response to TTFields. Further, we showed that TTFields combined with BTNPs exhibited antitumor activity by modulating several cancer-related pathways in general, and the cell cycle-related apoptosis pathway in particular. Therefore, our data suggest that BTNPs increase the antitumor action of TTFields by an electric field-responsive cytosolic accumulation, establishing BTNP as a TTFields-responsive sensitiser.

## Introduction

Tumour-treating fields (TTFields), a novel physical treatment modality approved by the US Food and Drug Administration (FDA), is known to be effective for solid therapy-resistant primary and recurrent tumours^1–3^. TTFields deliver alternating electric fields of low intensity (1–3 V/cm) and intermediate frequency (100–300 kHz) through non-invasive transducer arrays across the anatomical region of a tumour^4,5^. TTFields disrupts dipole alignments and induce dielectrophoresis^3–5^, and therefore, can preferentially inhibit proliferating cancer cells by interrupting polymerisation of mitotic microtubules and their assembly with polar particles during mitosis which leads to mitotic cell death^4–7^. Notably, TTFields do not affect the viability of non-dividing normal cells, nerves, and muscles because of their low intensity, frequency specificity, and loco-regional mode of application^3,5,8^. TTFields treatment, in combination with temozolomide, has been approved by FDA for newly diagnosed glioblastoma (GBM)^2,3^. Many preclinical and clinical studies indicate that TTFields would be applicable for other tumour types including breast, lung, pancreatic, and ovarian cancers^4,6,7,9,10^. Early clinical trials have shown that only TTFields treatment for GBM patients was not significantly better than conventional chemotherapy^3,9,11–13^. However, recent preclinical studies suggest that combination therapy of TTFields with conventional treatments including chemotherapy, immunotherapy, and radiotherapy are more effective than TTFields monotherapy in GBM^1,2,9,11–18^. Despite the promise shown by TTFields as a viable cancer therapy, not much is known about TTFields responsive sensitiser.

Ferroelectric nanomaterials have emerged as promising tools for enhancing electric stimulation of cells and tissues^19–23^. Several nanotransducers have been revealed to mediate photodynamic and magnetothermal conversions, and to locally deliver anticancer stimuli to tumour burden in the field of nanooncology^23^. Cell and tissue penetration of these nanotransducers could be controlled by remote electrical stimulation^22^. Among ferroelectric materials, barium titanate nanoparticles (BTNPs) have high dielectric constants and suitable piezoelectric characteristics with high biocompatibility^24^. Notably, recent reports suggest that BTNPs could be used in a wide range of applications in nanomedicine, including non-linear imaging purposes, drug delivery, tissue engineering, and bio-stimulation^19–22^. For instance, BTNPs promote higher internalisation of doxorubicin in human neuroblastoma cells^19^ and BTNPs with polyethylenimine have been shown to improve cellular uptake for cell imaging and DNA delivery^19^. In this backdrop, this study investigated whether BTNPs could enhance the antitumor action of TTFields in response to TTFields. Our data showed that BTNPs alone are cytocompatible with breast cancer cells, but in response to TTFields, it can sensitise TTFields-resistant breast cancer cells to the antitumor action of TTFields. Further, we demonstrated that BTNPs were taken up by TTFields stimulation and these promoted antitumor action of TTFields by enhancing cell cycle-related apoptosis in breast cancer cells. Therefore, this study constitutes the first report of a TTFields-responsive sensitiser, BTNPs, in breast cancer cells.

## Results

### Characteristics and cytocompatibility of BTNPs in breast cancer cells

**Figure 1:**
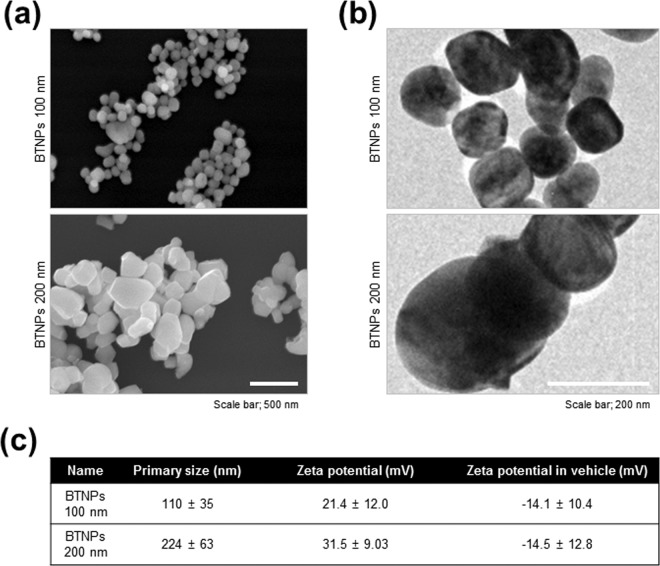
Physicochemical characterisation of BTNPs. (**a**) FE-SEM and (**b**) TEM images of BTNPs. (**c**) Sizes and zeta-potential values of FBS coated BTNPs.

**Figure 2:**
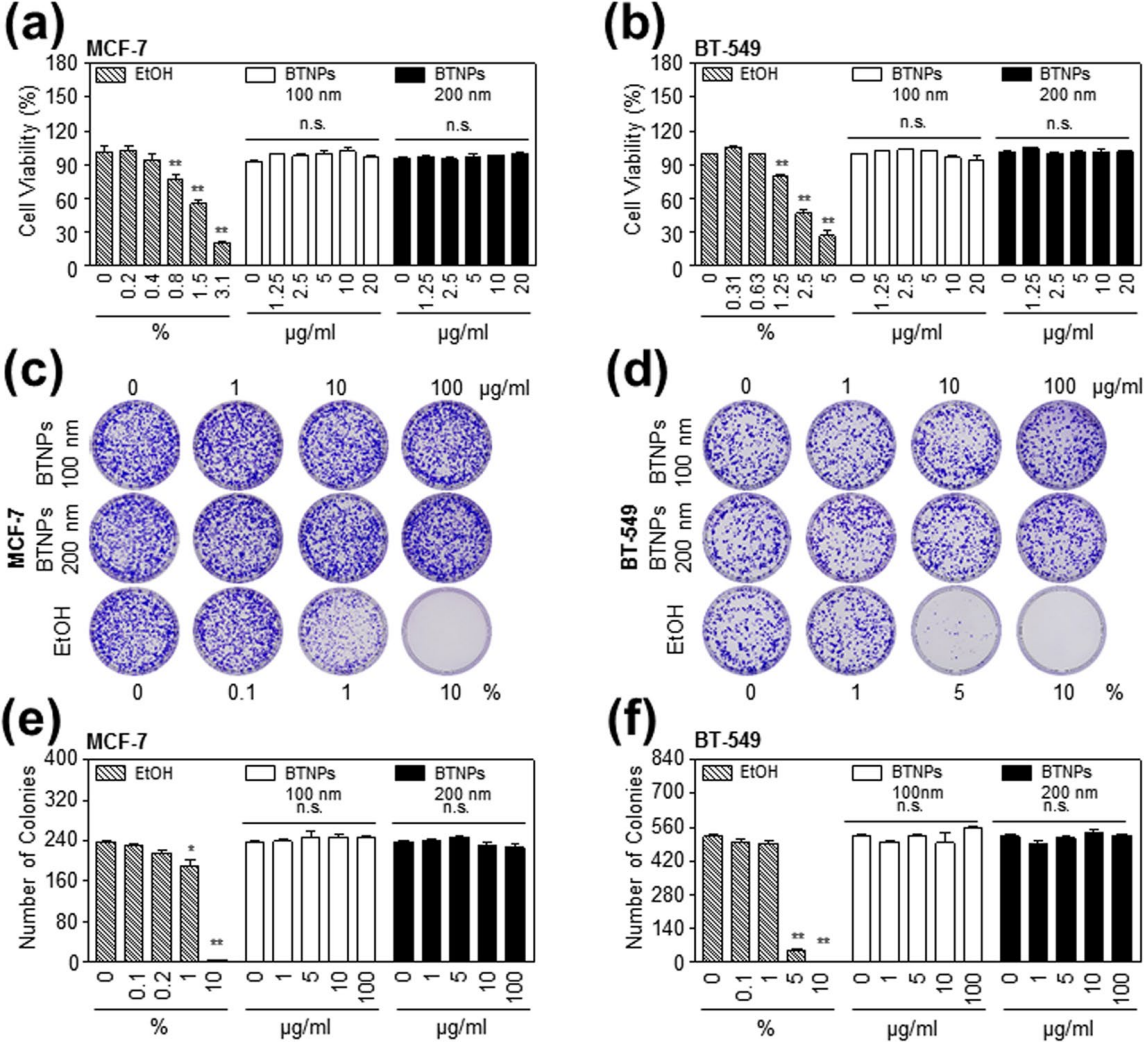
Cytocompatibility of BTNPs in breast cancer cells. (**a,b**) Cell proliferation, (**c,d**) representative images from clonogenic assays, and (**e,f**) colony counting in MCF-7 and BT-549 cells upon BTNP treatment. Data represent mean ± standard deviation of three independent experiments; **P < 0.01, and *P < 0.05. N.S. not significant.

Since dielectric permittivity of BTNPs can be maximised depending on its size^25,26^, we prepared two different sizes of FBS (foetal bovine serum) coated BTNPs (100 nm and 200 nm). The SEM images of 100 nm and 200 nm BTNPs showed typical round shape and homogeneous size of the nanoparticles (Fig. 1a,b). The hydrodynamic radius of 100 nm and 200 nm BTNPs were 110 ± 35 nm and 224 ± 63 nm, respectively. The measured zeta potential values on the original surface of 100 nm and 200 nm BTNPs were 21.4 ± 12.0 mV and 31.5 ± 9.0 mV, respectively, and the values were −14.1 ± 10.4 mV and −14.5 ± 12.8 mV, respectively after attachment of FBS to BTNPs (Fig. 1c), indicating that BTNPs were relatively stable in aqueous dispersions. Next, the cytocompatibility of 100 nm and 200 nm BTNPs were examined by cell viability and clonogenic assay in the two breast cancer cell lines, MCF-7 and BT-549. Ethanol was used as a positive control in these assays. The cell viability assay indicated that treatment with 100 nm and 200 nm BTNPs up to a concentration of 20 μg/ml did not affect cell viability in MCF-7 and BT-549 cells (Fig. 2a,b). In addition, the clonogenic assay showed that treatment with 100 nm and 200 nm BTNPs up to a concentration of 100 μg/ml did not affect colony formation in MCF-7 and BT-549 cells (Fig. 2c–f). Moreover, BTNPs without FBS coating did not affect cell viability in MCF-7 and BT-549 cells (Supplementary Fig. S1). Taken together, these results suggest that BTNPs exhibit cytocompatibility, with non-cytotoxic effects in breast cancer cells.

### BTNPs sensitise TTFields-resistant breast cancer cells in response to TTFields

**Figure 3:**
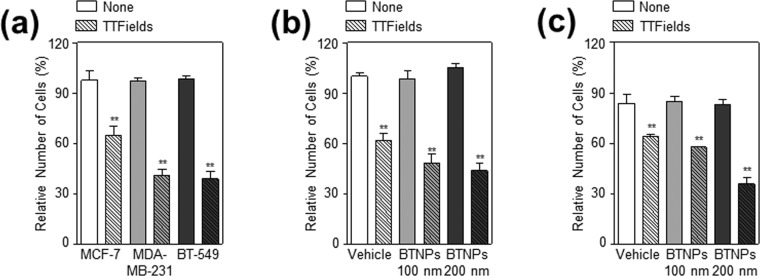
BTNPs enhanced the antitumor activity of TTFields in TTFields-resistant MCF-7 cells. (**a**) Cell proliferation following TTFields in MCF-7, MDA-MB-231, and BT-549 cells, (**b**) Relative number of cells with TTFields or TTFields and BTNPs treatment to MCF-7 cells, and (**c**) quantification of colonies. Data represent mean ± standard deviation of five independent experiments; **P < 0.01, and *P < 0.05.

Since it has been reported that the efficacy of TTFields is different across different cancer cell lines^4,7^, TTFields efficacy were tested in three breast cancer cell lines, MCF-7, MDA-MB-231, and BT-549. Among these, MCF-7 cells were more resistant to TTFields than the other two breast cancer cell lines (Fig. 3a), which is consistent with a previous report^7^. Thus, the combinatorial effect of BTNPs and TTFields was examined in MCF-7 cells. Cell viability and clonogenic assays showed that treatment with 100 nm and 200 nm BTNPs enhanced the antitumor action of TTFields in TTFields-resistant MCF-7 cells (Fig. 3b,c). Notably, 200 nm BTNPs were more potent than the 100 nm ones (Fig. 3b,c), suggesting that size may be an important factor in the antitumor activity of BTNPs in presence of TTFields. Thus, these results indicated that BTNPs sensitise TTFields-resistant breast cancer cells in response to TTFields.

### TTFields induce the cytosolic accumulation of BTNPs in breast cancer cells

**Figure 4:**
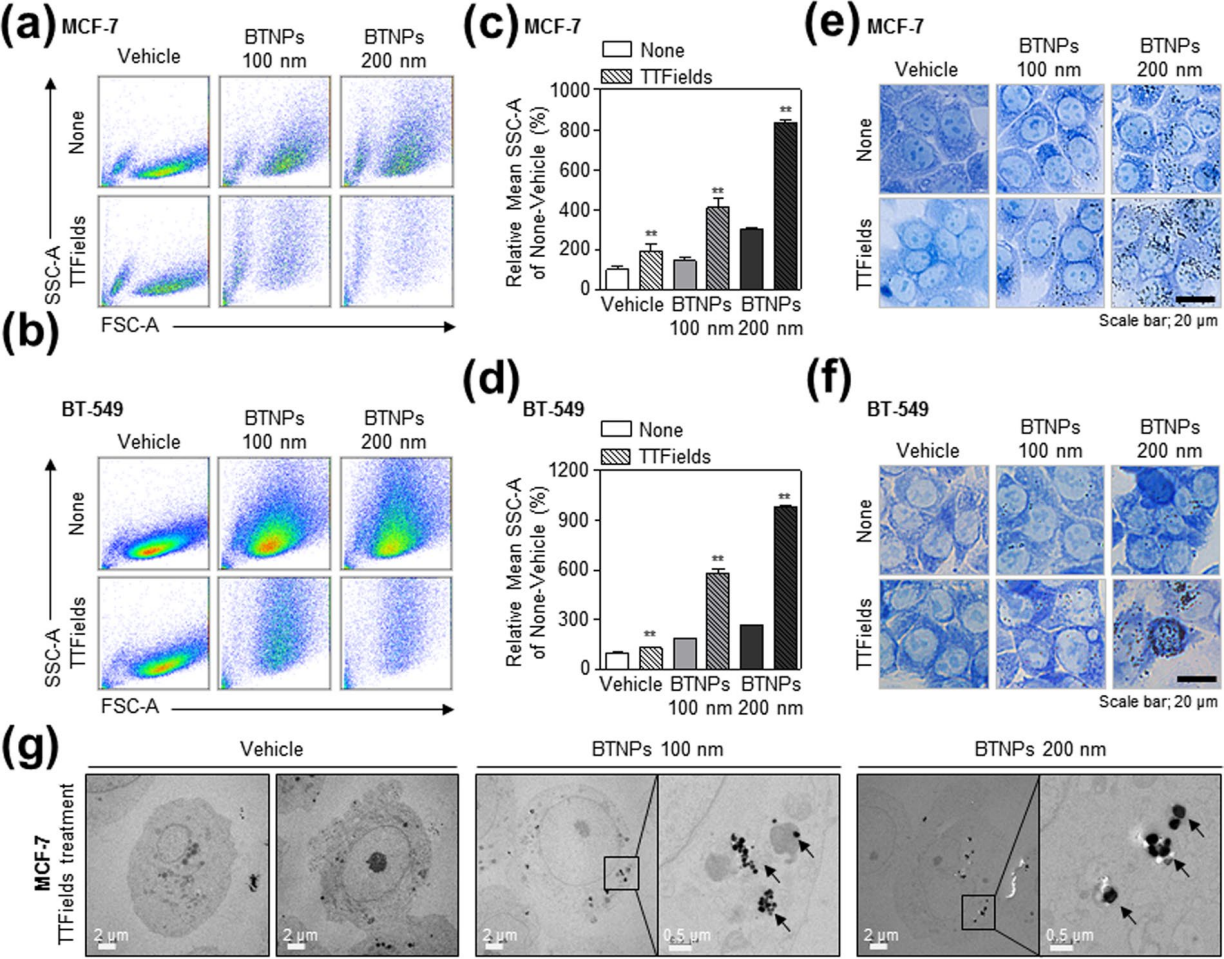
Cytoplasmic accumulation of BTNPs in MCF-7 and BT-549 cells in response to TTFields. (**a,b**) Flow cytometry histogram, (**c,d**) relative granularity, and (**e,f**) representative images showing cytosolic localisation of BTNPs in MCF-7 and BT-549 cells treated with TTFields or TTFields and BTNP. (**g**) TEM images confirming the cytosolic localisation of BTNPs in TTFields-treated MCF-7 cells. Data is representative of three independent experiments.

To investigate the mechanism of this sensitisation mediated by BTNPs in presence of TTFields, we next examined whether BTNPs accumulate into breast cancer cells in response to TTFields. First, we performed a fluorescence-activated cell sorting (FACS) analysis to determine cell size and granularity in TTFields-treated and BTNP/TTFields-treated cells. These parameters were similar between the control and TTFields-treated MCF-7 and BT-549 cells (Fig. 4a–d). However, cell size and granularity increased in BTNP/TTFields-treated MCF-7 and BT-549 cells (Fig. 4a–d). In addition, bright fields images of cells stained with methylene blue showed the cytosolic accumulation of BTNPs in response to TTFields in MCF-7 and BT-549 cells (Fig. 4e,f). Notably, transmission electron microscopy (TEM) analysis showed that BTNPs accumulated in the cytoplasm of TTFields-treated MCF cells (Fig. 4g); these results indicate that BTNPs accumulated in the cytoplasm of breast cancer cells in response to TTFields.

### TTFields combined with BTNPs modulates cell cycle-apoptosis pathways

**Figure 5:**
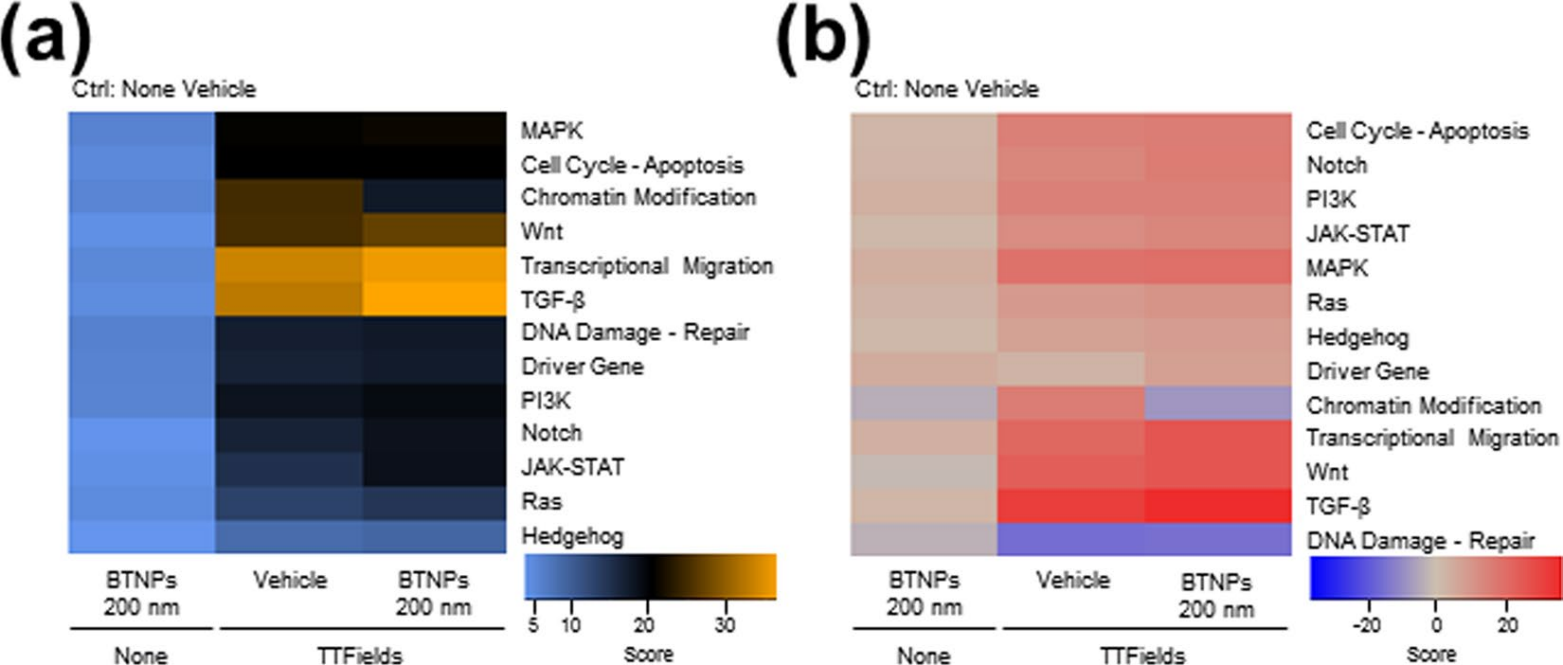
Changes in gene copy number in MCF-7 cells on TTFields and BTNP combinatorial treatment. (**a**) Heatmap with global significance scores and global significance statistics and (**b**) directed global significance scores for cells treated with TTFields or TTFields and BTNPs.

**Figure 6:**
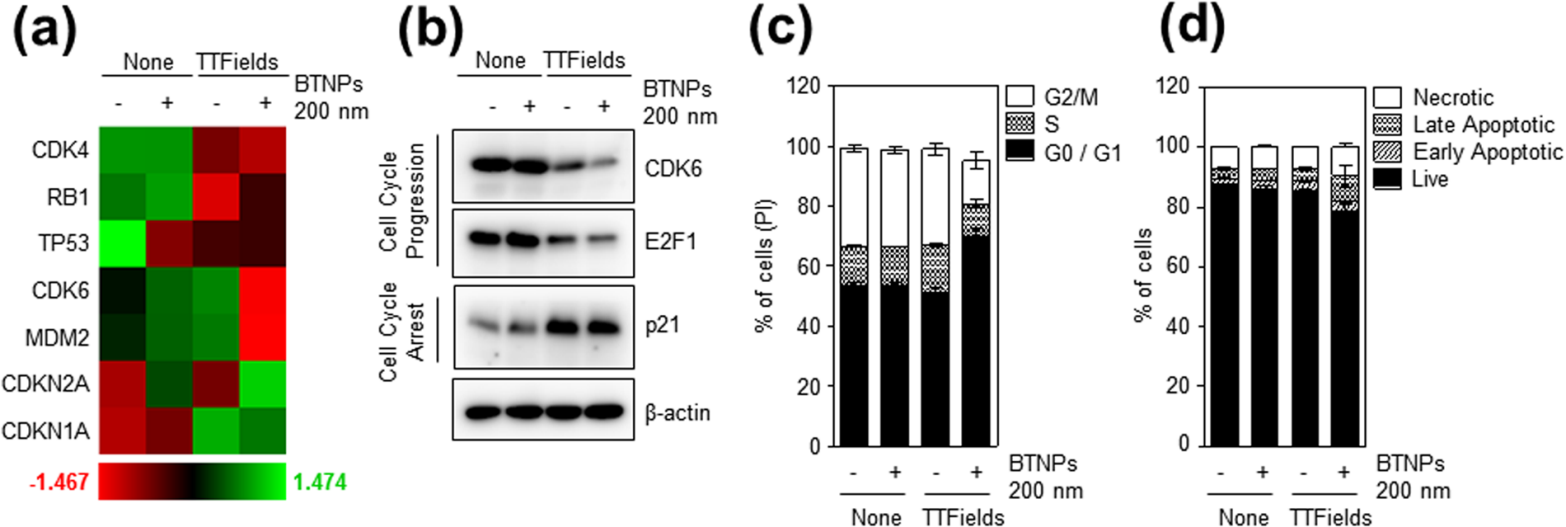
Modulation of cell cycle-apoptosis pathways by BTNPs combined with TTFields. (**a**) Gene signatures related to cell cycle pathways grouped in a heatmap and (**b**) Western blot of MCF-7 cells treated with TTFields or TTFields and BTNP. (**c**) Cell cycle and (**d**) apoptosis of MCF-7 cells treated with TTFields or TTFields and BTNP. Data is representative of three independent experiments. Blotting results in (**b**) were cropped from different gels and were therefore delineated by white spaces and lines. The original blot data is shown in Supplementary Fig. S4.

To further investigate the regulatory action of the TTFields/BTNPs combination approach, a NanoString nCounter^TM^ Pan-Cancer pathway analysis containing probes targeting 700 transcripts related to 13 types of cancer pathways was carried out in MCF-7 cells exposed to TTFields and treated without or with 200 nm BTNPs for 48 hrs. MCF-7 cells treated with BTNPs with no exposure to TTFields was also included as a control. Overall, the gene expression patterns were similar between control and BTNPs-treated MCF-7 cells, while TTFields treatment induced dramatic changes in 9 different types of cancer pathways (Fig. 5a). Among them, cell cycle-apoptosis, Wnt, transcriptional migration, transforming growth factor beta (TGF-β), driver gene, Notch, Janus kinase-signal transducer and activator of transcription (JAK-STAT), and Ras signalling were significantly modulated in TTFields-treated and BTNP/TTFields-treated MCF-7 cells (Fig. 5b), implying that BTNPs/TTFields have a capacity to modulate several cancer signalling pathways. As it is well established that TTFields disrupt mitosis of cancer cells^6,7,27^, the cell cycle pathways were further analysed. Interestingly, we found that several cell cycle regulatory transcripts including cyclin dependent kinase 4 (CDK4), RB1, tumour protein TP53, cyclin dependent kinase 6 (CDK6), MDM2, and CDKN1A/2 A were modulated in BTNP/TTFields-treated MCF-7 cells (Fig. 6a). In addition, Western blot analysis for the cell cycle regulatory genes also showed that TTFields combined with BTNPs inhibited cell cycle progression, as determined by a significant decrease in levels of CDK6 and transcription factor E2F1, both key regulators of G1 cell cycle progression^28^, and an increase in p21 levels, a key regulator of cell cycle arrest^28^ (Fig. 6b). Consistently, FACS analysis for cell cycle indicated that TTFields combined with BTNPs inhibited cell cycle progression by inducing cell-cycle arrest at G1 phase (Fig. 6c). Moreover, we observed that TTFields combined with BTNPs slightly increased apoptosis (Fig. 6d), implying that the combination induces cell cycle arrest rather than apoptosis. Therefore, our results suggest that TTFields combined with BTNPs exerts anticancer activity on breast cancer cells by modulating cancer-related pathways, and specifically inhibiting cell cycle progression.

## Discussion

Although there is an accumulating body of evidence demonstrating enhanced efficacy of the combined treatment of TTFields and chemotherapeutic agents or radiotherapy^1,2,9,11–18,29^, not much is known about TTFields-responsive sensitisers. Here, we report novel TTFields-responsive sensitisers, BTNPs, characterised by high dielectric constants. We demonstrated that BTNPs had non-cytotoxic effects in breast cancer cells and enhanced the antitumor activity of TTFields-resistant breast cancer cells in response to TTFields. Further, we found that TTFields triggered the accumulation of BTNPs, which promoted the cell cycle-related apoptosis pathway. Therefore, our study provides the first evidence that biocompatible nanomaterials such as BTNPs could be used as TTFields-responsive sensitiser in cancer cells.

Our results showed that BTNPs had non-cytotoxic effects even at high concentrations (100 μg/ml) in breast cancer cells, suggesting that these are biocompatible. Consistent with our results, other reports have shown that treatment with BTNPs have minimal adverse effects, as evident from several assays including metabolic activity, viability/cytotoxicity, early apoptosis, and reactive oxygen species (ROS) generation in multiple types of cells such as human neuroblastoma SH-SY5Y cells^19^, HeLa cells^20^, and rat mesenchymal stem cells^30^. Indeed, we also observed that treatment with only BTNPs did not significantly alter the 13 types of major cancer pathways and cell cycle regulatory proteins (Fig. 5). In addition, several studies indicated that polymeric coated BTNP have increased stability in aqueous solutions^21^. For instance, poly-L-lysine- or glycol-chitosan-coated BTNPs efficiently stabilised BTNPs in an aqueous solution and exhibited low cytotoxicity^31^. Therefore, BTNPs and its coated composites could be used as a biocompatible sensitiser for TTFields.

It is reported that TTFields efficacy is dependent on cell doubling time in various cancer cell lines^7^. However, we observed that MCF-7 cells were more resistant to TTFields than MDA-MB-231 and BT-549 cells, despite the similar cell doubling time between MCF-7 and MDA-MB-231 cells^7^. Similarly, a recent study showed that non-small cell lung cancer cell lines had different responsiveness against TTFields depending on *BRCA1* pathway regardless of its doubling time^10^, suggesting that the cell doubling time as well as genetic background of cancer cells may be associated with tumour resistance to TTFields.

We observed that BTNPs were accumulated into the cytoplasm of breast cancer cells in response to TTFields. Nanoparticles (NPs) internalisation into cells is known to be dependent on particle size and its zeta potential^32^. NPs under 200 nm can be engulfed by cancer cells through clathrin-dependent pathway or macro-pinocytosis pathway^32,33^. However, we observed that specific inhibitors for these pathways such as amiloride and cytochalasin D did not modulate the accumulation of BTNPs in cytoplasm in response to TTFields (Supplementary Fig. S2), suggesting that BTNP accumulation in cytoplasm is not mediated by clathrin-dependent pathway or macro-pinocytosis pathway. Instead, a recent study showed that TTFields have a capacity to induce membrane pores in glioblastoma cells, which may allow cancer cells to be susceptible to drug delivery^34^. Therefore, it seems that increased membrane permeability by TTFields may induce BTNP accumulation in cytoplasm of cancer cells.

In addition, we observed that 200 nm BTNPs were more potent in terms of antitumor activity than the 100 nm ones. This may be associated with the difference in cytosol accumulation between 100 nm and 200 nm BTNPs, since 200 nm BTNPs showed higher accumulation in the cytoplasm than the 100 nm ones (Fig. 4). Another possibility is that a smaller size of BTNPs could decrease their dielectric permittivity^25,26^. Indeed, we observed that the 200 nm BTNPs had a higher dielectric constant than the 100 nm BTNPs due to the higher average grain size value, obtained from the X-ray diffraction data using the Scherrer formula (Supplementary Fig. S3), suggesting that size may be an important factor in the antitumor activity of BTNPs in presence of TTFields.

We found that TTFields combined with BTNPs modulated the cell cycle-apoptosis pathways using NanoString nCounter analysis. It is well established that TTFields induces mitotic arrest by interrupting polymerisation of mitotic microtubules during mitosis, thereby leading to mitotic cell death^4–7^. Consistently, our data indicated that TTFields combined with BTNPs significantly modulated the cell cycle-apoptosis pathways over other related pathways. Since cells with mitotic defects undergo mitotic catastrophe or G1-arrest senescence, our data may imply that TTFields combined with BTNPs could induce mitotic catastrophe and G1-arrest senescence by modulating cell cycle-apoptosis pathway, as evident by the decrease in G1 cell cycle regulators including CDK4/6, p-RB, and E2F1 in the BTNPs/TTFields-treated cells. In addition to cell cycle-apoptosis pathway, we also observed significant modulation of several cancer pathways including Wnt, transcriptional migration, transforming growth factor beta (TGF-β), driver gene, Notch, Janus kinase-signal transducer and activator of transcription (JAK-STAT), and Ras signalling in TTFields-treated and BTNPs/TTFields-treated MCF-7 cells. So far, very few reports exist on the role of TTFields in the regulation of these pathways in cancer cells. Therefore, further explorations are required to understand the role of TTFields in the regulation of several cancer pathways.

**Figure 7:**
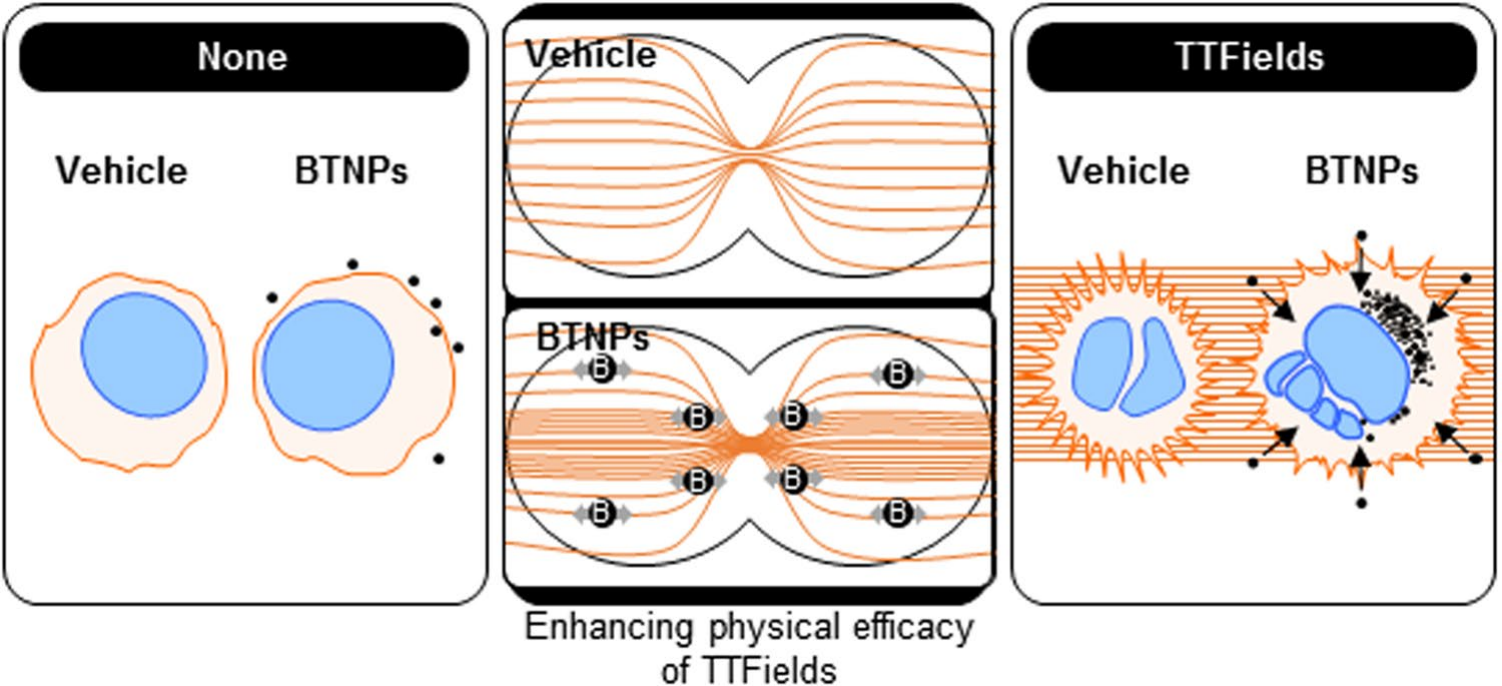
Schematic representation of the proposed mechanism of cancer cell sensitisation induced by BTNPs in presence of TTFields.

In summary, our data showed that BTNPs, characterised by their high biocompatibility and ferroelectric properties, acts as a TTFields-responsive sensitiser to breast cancer cells by modulating cell cycle-apoptosis pathway (Fig. 7). Therefore, our work has demonstrated, for the first time, that electric field responsive nanomaterials such as BTNPs could be used as a TTFields-responsive sensitiser to enhance the therapeutic efficacy of TTFields in cancer cells.

## Materials and Methods

### Cell culture

MCF-7, BT-549, and MDA-MB-231 breast cancer cell lines were purchased from American Type Culture Collection (ATCC, Manassas, VA). As confirmed by the information provided by ATCC, both cell lines were authenticated by their karyotypes, images, and detailed gene expression. Both cell lines were preserved and passaged in less than 2 months in accordance with ATCC protocols, and tested for mycoplasma infection by polymerase chain reaction (PCR) once a week. MCF-7 cells were cultured in Dulbecco’s Modified Eagle Media (DMEM, Corning, NY, USA). BT-549 and MDA-MB-231 cells were cultured in RPMI (Corning, NY, USA). All media types were supplemented with 10% foetal bovine serum (FBS, Corning, NY, USA) and 1% penicillin/streptomycin (Sigma-Aldrich, MO, USA). All cell lines were maintained in a humidified 5% CO_2_ incubator at 37 °C.

### TTFields application

MCF-7 (1.5 × 10^4^), BT-549 (1 × 10^4^), and MDA-MB-231 (3 × 10^4^) cells were seeded on 18 mm glass coverslips (Marienfeld-Superior, Mediline, Lauda-Königshofen, Germany) or 22 mm plastic coverslips (Thermo Fisher Scientific, MA, USA) for 24 hrs and those coverslips were transferred to ceramic inovitro dishes (NovoCure, Haifa, Israel) using autoclaved forceps. For TTFields treatment, we applied the inovitro™ system (NovoCure, Haifa, Israel) for 72 hrs as described previously^6,7^. Briefly, cells on a coverslip were exposed to 2 V/cm at 150 kHz with a current of 150 mA generated by inovitro TTFields generators (NovoCure, Haifa, Israel) and the plate temperature was maintained at 37 °C by a refrigerated incubator (ESCO Technologies, USA) at 19 °C.

### Generation and physicochemical characterisation of BTNPs

Barium titanate nanoparticles (100 nm, 200 nm) were purchased from US Research Nanomaterials Inc. (TX, USA) and used without further purification. BTNPs were dispersed in ethanol and sonicated to mitigate aggregation. In addition, 5% FBS was added to coat the surface of BTNPs with a protein corona, before addition to cells. The nanostructures and morphologies of prepared BTNPs were examined by field emission scanning electron microscopy (FE-SEM) with a Sirion-400 (FEI, OR, USA) and TEM with a JEM-2100F (JEOL, Japan). The zeta-potential of FBS coated BTNPs were measured by dynamic light scattering in a Zetasizer Nano ZS (Malvern Instruments Ltd., UK). X-ray diffraction patterns were measured using a D/MAX-2500 (Rigaku, Japan).

### Cell viability assay

Cell viability assays were performed using WST-8 reagent (Cyto X; LPS solution, Daejeon). MCF-7 cells (0.5 × 10^4^) were seeded on a 96-well plate and treated with media containing increasing concentrations of BTNPs or ethanol as a vehicle control. After 72 hrs, WST-8 reagent (10 μl) was added to each well and the plate incubated for 2 hrs at 37 °C. Subsequently, the absorbance was measured at 450 nm using a VersaMax Microplate Reader (Molecular Devices, CA, USA).

### Clonogenic assay

The clonogenic assay was performed as described previously^7,10,35^. MCF-7 or BT-549 cells (500 in number) were seeded on a 22 mm plastic coverslip in a 6-well plate for 24 hrs. Using autoclaved forceps, the coverslips were transferred to ceramic inovitro dishes and incubated with inovitro TTFields generators for 72 hrs. After TTFields treatment, the coverslips were transferred to a 6-well plate and incubated at 37 °C. After 7 days, colonies were fixed and stained with 1% crystal violet (Sigma-Aldrich) and 40% methanol solution, and the number of colonies counted.

### FACS analysis

To evaluate the number of alive cells in the same volume, absolute cell counts were acquired using a BD Accuri™ C6 flow cytometer (BD Biosciences, CA, USA) as described previously^35^. Briefly, detached MCF-7 and BT-549 cells in fresh media (500 μl) were stained with propidium iodide (50 μg/ml; PI; Sigma-Aldrich, MO, USA) and the number of cells in PI-negative population was counted in a 100 μl volume. To investigate the accumulation of BTNPs in MCF-7 and BT549 cells, the cell population abundance was analysed within FSC-A and SSC-A plots. After this, mean SSC-A value in PI-negative population was quantified and relative Mean SSC-A values were calculated based on none-vehicle. Cell cycle analysis was performed following the method previously described^35^. Briefly, the cells treated with TTFields or TTFields and BTNP for 72 hrs were trypsinized, washed twice in PBS, and fixed with ice-cold 70% ethanol. Fixed cells were incubated with 50 μg/mL PI and 100 μg/mL RNase for 30 min at 37 °C and then analyzed with BD accuri™ C6.

### Apoptosis analysis

MCF-7 cells were treated with TTFields or TTFields and BTNP. After 72 hrs, apoptosis assays were performed using FITC Annexin V Apoptosis Detection Kit (BD biosciences, CA, USA) following the manufacture’s protocol. The samples were analyzed using BD Accuri C6 flow cytometer.

### Methylene blue staining

Cells were fixed with 4% paraformaldehyde, and then stained with 0.1% methylene blue (Sigma-Aldrich, MO, USA) dissolved in Dulbecco’s phosphate-buffered saline (DPBS) for 5 min. After washing several times with DPBS, the slides were mounted in glycerol and images obtained using an LSM 710 confocal microscope (Carl Zeiss Inc., Germany).

### TEM imaging

TTFields treated MCF-7 cells with and without BTNP treatment were detached and fixed in 2.5% glutaraldehyde (Sigma-Aldrich, MO, USA) and 0.1 M phosphate buffer (pH 7.3) at 4 °C overnight. After this fixation, the cells were treated with 1% osmium tetroxide and 1.5% potassium ferrocyanide in 0.1 M phosphate buffer (pH 7.3) for 1 h at 4 °C in dark. Subsequently, these were embedded in Epon 812 (Sigma-Aldrich, MO, USA) after dehydration in a treatment cycle of ethanol and propylene oxide. The polymer reaction was carried out by using pure resin at 70 °C for two days. Ultrathin samples were obtained with an UltraCut-UCT ultramicrotome (Leica, Austria) and collected on 150 mesh copper grids. After staining with 2% uranyl acetate for 10 min and lead citrate for 5 min, the samples were examined at 120 kV in a Tecnai G2 Spirit Twin TEM setup (FEI, OR, USA, installed at Korea Basic Science Institute).

### RNA isolation and NanoString analysis

MCF-7 cells were treated with indicated conditions. After 72 hrs, total RNA was isolated using QIAzol reagents (Qiagen, Hilden, Germany) from treated cells. Following the procedures provided by the nCounter XT CodeSet Gene Expression Assays (NanoString Technologies, WA, USA), 100 ng of RNA was used to hybridise with probes.

### Western blot analysis

Western blotting was performed as described previously^35,36^. Briefly, proteins were separated by SDS-polyacrylamide gel electrophoresis, transferred to a nitrocellulose membrane, and detected using specific antibodies. The following antibodies were used: rabbit monoclonal CDK6 (Santa Cruz Biotechnology, CA, USA); mouse monoclonal p21, mouse monoclonal E2F1, and mouse polyclonal anti-β-actin (Santa Cruz Biotechnology, CA, USA). Blots were developed using peroxide-conjugated secondary antibody and visualised with an enhanced chemiluminescence detection system (Amersham Life Science, Buckinghamshire, UK).

### Statistical analysis

The two-tailed Student’s *t*-test was performed to analyse statistical differences between groups. *P*-values of less than 0.05 were considered statistically significant. Statistical analyses were performed using Microsoft Excel and XLSTAT software.

## Supplementary information

Supplementary Information.

## Data Availability

The datasets generated and analysed during the current study are available from the corresponding author on reasonable request.
