# Supplementary material for: Barium Titanate Nanoparticles Sensitise Treatment-Resistant Breast Cancer Cells to the Antitumor Action of Tumour-Treating Fields
Source: Sci Rep. 2020 Feb 13;10:2560. doi: 10.1038/s41598-020-59445-x (PMC7018996; doi:10.1038/s41598-020-59445-x)

**Supplementary Information**

# Barium titanate nanoparticles sensitise treatment-resistant breast cancer cells to the antitumor action of tumour-treating fields

Yi Na Yoon[a,b,§], Dae-Sik Lee[c,§], Hyung Ju Park[c,]*, and Jae-Sung Kim[a,b,]*

[a]Division of Radiation Biomedical Research, Korea Institute of Radiological and Medical Sciences, Seoul 01812, South Korea
[b]Radiological and Medico-Oncological Sciences, University of Science and Technology, Daejeon 34113, South Korea
[c]Electronics and Telecommunications Research Institute, Daejeon 34129, South Korea

[§]Y.N.Y. and D.-S.L. contributed equally to the work

***Corresponding author**: Jae-Sung Kim, Division of Radiation Biomedical Research, Korea Institute of Radiological and Medical Sciences, 75, Nowon-ro, Nowon-Ku, Seoul 01812, South Korea. Phone: +82-2-970-1669, Fax: +82-2-970-2417, E-mail: jaesung@kirams.re.kr or Hyung Ju Park, Welfare & Medical ICT Research Department, Electronics and Telecommunications Research Institute (ETRI), 218 Gajeong-ro, Yuseong-gu, Daejeon, 34129, South Korea. Phone: +82-42-860-1474, Fax: +82-42-860-6594, E-mail: park77@etri.re.kr

# Supplementary Figure

**Supplementary figure S1. Cytocompatibility of BTNPs in breast cancer cells.** (a-d) Cell proliferation in MCF-7 and BT549 cells upon BTNP treatment with or without FBS-coating. Data represent mean ± standard deviation of three independent experiments; ***P* < 0.01, and **P* < 0.05. N.S. not significant.

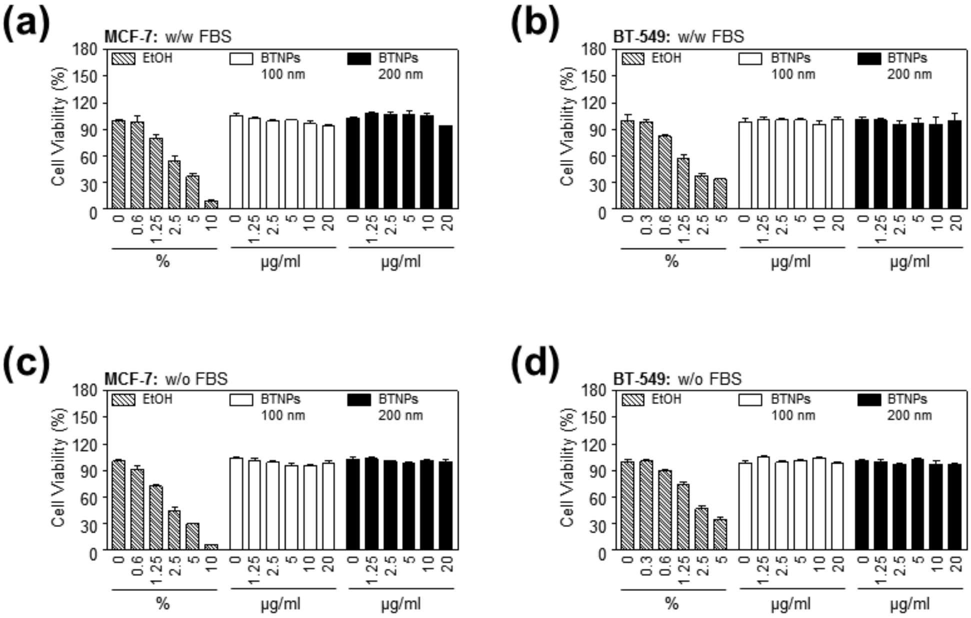

**Supplementary figure S2. Cytoplasmic accumulation of BTNPs with inhibitors in MCF-7 cells in response to TTFields.** (a) Schematic summary of the experiment. (b) Representative images showing cytosolic localisation of BTNPs in MCF-7 cells treated with TTFields or TTFields plus BTNPs without or with cytochalasin D and amiloride. Data is representative of three independent experiments.

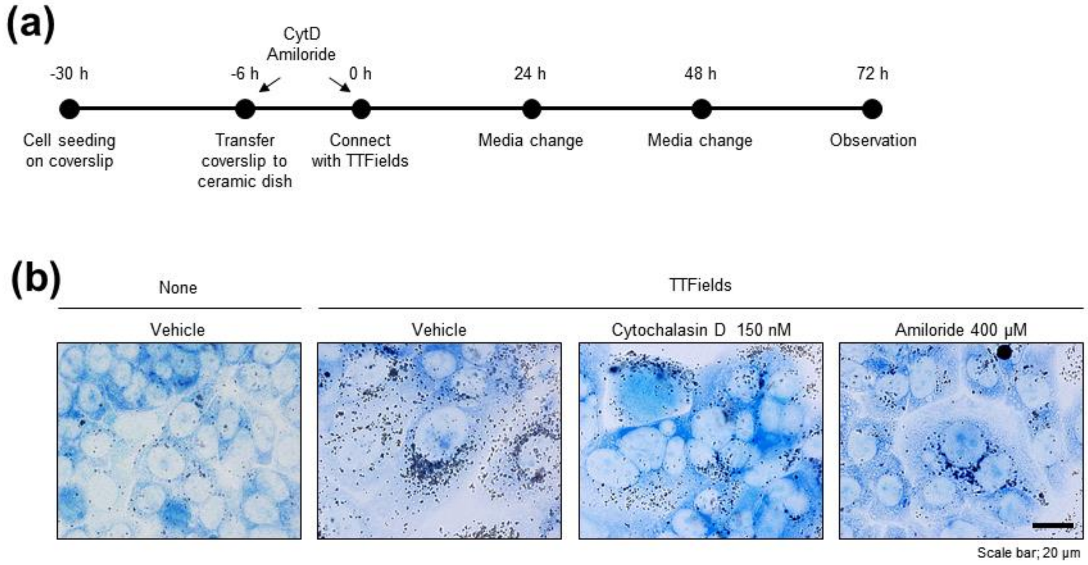

**Supplementary figure S3. XRD spectral changes of $BaTiO_3$ nanoparticles. (a) Schematic summary of the experiment.**

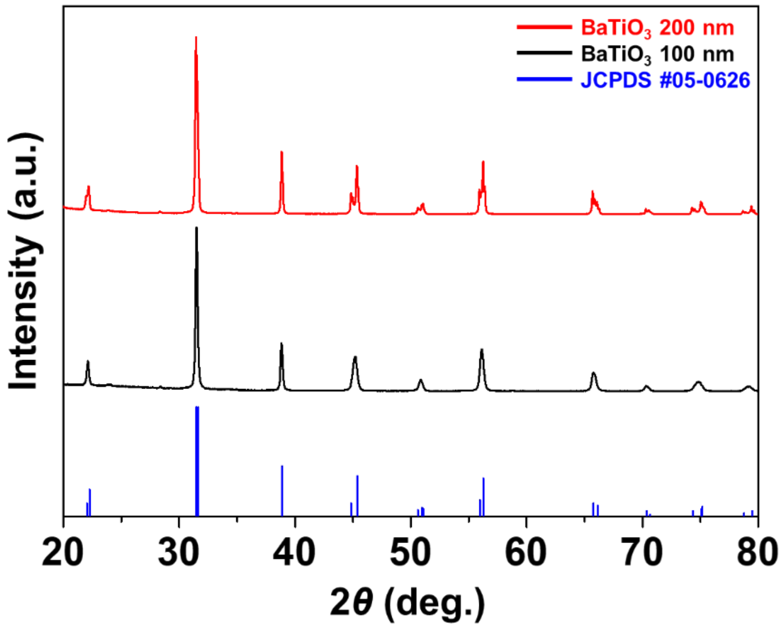

**Supplementary figure S4.** Original blots used for Figure 6 (b).

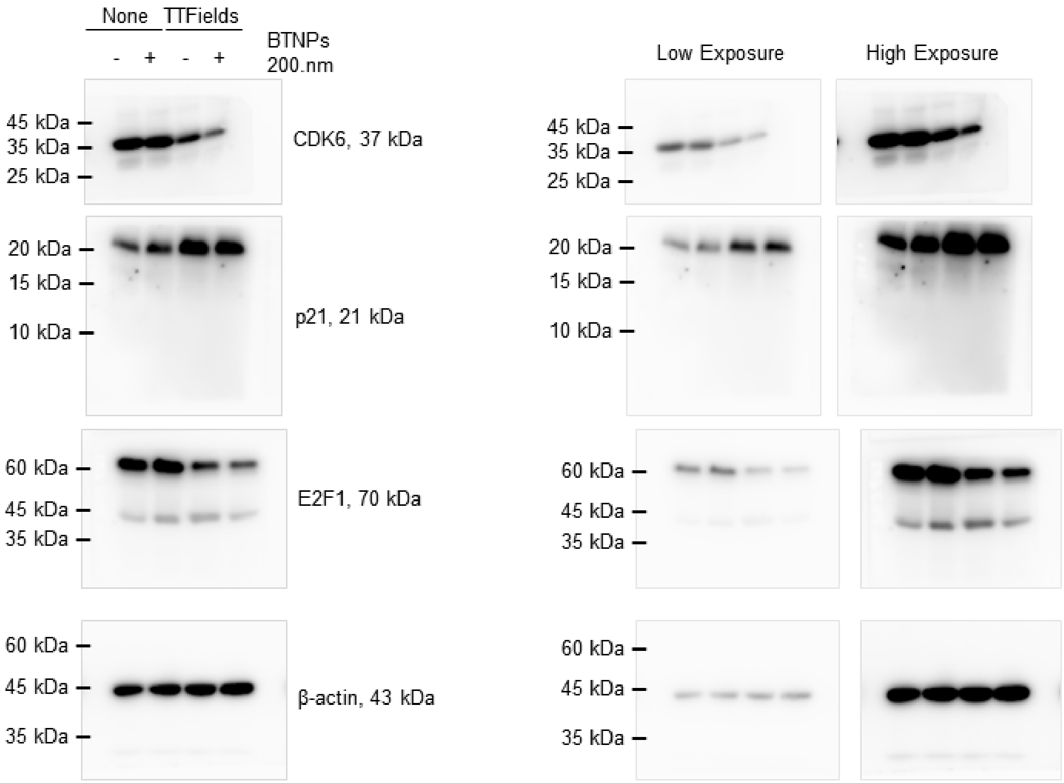

Supplement: Supplementary file 1 — Supplementary Information. [file 41598_2020_59445_MOESM1_ESM.pdf]
